# Low caveolin‐1 levels and symptomatic intracranial haemorrhage risk in large‐vessel occlusive stroke patients after endovascular thrombectomy

**DOI:** 10.1111/ene.16342

**Published:** 2024-05-17

**Authors:** Yi Xie, Min Wu, Yun Li, Ying Zhao, Shuaiyu Chen, E. Yan, Zhihang Huang, Mengdi Xie, Kang Yuan, Chunhua Qin, Xiaohao Zhang

**Affiliations:** ^1^ Department of Neurology Affiliated Jinling Hospital, Medical School of Nanjing University Nanjing China; ^2^ Department of Neurology Jinling Hospital, Nanjing Medical University Nanjing China; ^3^ Department of Neurology Nanjing First Hospital, Nanjing Medical University Nanjing China

**Keywords:** caveolin‐1, endovascular treatment, haemorrhage, ischaemic stroke

## Abstract

**Background and purpose:**

Caveolin‐1 (Cav‐1) is reported to mediate blood–brain barrier integrity after ischaemic stroke. Our purpose was to assess the role of circulating Cav‐1 levels in predicting symptomatic intracranial haemorrhage (sICH) amongst ischaemic stroke patients after endovascular thrombectomy (EVT).

**Methods:**

Patients with large‐vessel occlusive stroke after EVT from two stroke centres were prospectively included. Serum Cav‐1 level was tested after admission. sICH was diagnosed according to the Heidelberg Bleeding Classification.

**Results:**

Of 325 patients (mean age 68.6 years; 207 men) included, 47 (14.5%) were diagnosed with sICH. Compared with patients without sICH, those with sICH had a lower concentration of Cav‐1. After adjusting for potential confounders, multivariate regression analysis demonstrated that the increased Cav‐1 level was associated with a lower sICH risk (odds ratio 0.055; 95% confidence interval 0.005–0.669; *p* = 0.038). Similar results were obtained when Cav‐1 levels were analysed as a categorical variable. Using a logistic regression model with restricted cubic splines, a linear and negative association of Cav‐1 concentration was found with sICH risk (*p* = 0.001 for linearity). Furthermore, the performance of the conventional risk factors model in predicting sICH was substantially improved after addition of the Cav‐1 levels (integrated discrimination index 2.7%, *p* = 0.002; net reclassification improvement 39.7%, *p* = 0.007).

**Conclusions:**

Our data demonstrate that decreased Cav‐1 levels are related to sICH after EVT. Incorporation of Cav‐1 into clinical decision‐making may help to identify patients at a high risk of sICH and warrants further consideration.

## INTRODUCTION

Endovascular thrombectomy (EVT) is the most effective approach for large‐vessel occlusion in the anterior circulation within 24 h from stroke onset, as substantiated by randomized controlled trials [1, 2]. Despite achieving arterial recanalization and subsequent reperfusion, more than half of stroke patients continue to experience unfavourable outcomes [3, 4]. Symptomatic intracranial haemorrhage (sICH), the most serious complication of EVT, could result in early neurological deterioration and functional impairment [5, 6, 7]. There remains uncertainty regarding the factors that may impact the development of sICH. The delineation of specific predictors of sICH could affect periprocedural management, particularly in evaluating the need for intensive care after mechanical thrombectomy.

Caveolin‐1 (Cav‐1) is a 22 kDa coat protein of caveolae, regulating various cell signalling pathways [8]. Cav‐1 has been reported to be highly expressed in brain vascular endothelium [9, 10, 11]. The present authors and others have revealed a critical role of Cav‐1 in maintaining blood–brain barrier integrity and endothelial homeostasis after cerebral ischaemia [12, 13, 14]. According to the data from the Nanjing Stroke Registry Programme, serum Cav‐1 level could predict the presence of cerebral microbleeds in ischaemic stroke patients [15], indicating that Cav‐1 might be a potential predictor of bleeding events in ischaemia. Furthermore, decreased levels of Cav‐1 were associated with sICH after recombinant tissue‐type plasminogen activator administration [16]. However, the relationship between Cav‐1 levels and sICH remains largely unknown in stroke patients treated with EVT.

In the present study, whether circulating Cav‐1 level could predict the risk of sICH in a cohort of ischaemic stroke patients receiving EVT treatment was investigated.

## METHODS AND MATERIALS

### Study design and population

From September 2019 to July 2021, consecutive patients with anterior circulation large‐vessel occlusion and EVT were prospectively included from two university‐affiliated hospitals (Nanjing First Hospital and Jinling Hospital). EVT treatment was performed based on the protocols of each hospital, in adherence to the most recent EVT guidelines. Inclusion criteria to screen patients were (i) age >18 years old; (ii) ischaemic stroke with anterior circulation large‐vessel occlusion (internal carotid artery, M1/M2 segment of middle cerebral artery), which was confirmed by computed tomography angiography, magnetic resonance angiography or digital subtracted angiography. The following patients were excluded: (i) those who were treated with devices other than a stent‐like retriever and aspiration system or treated with intra‐arterial thrombolysis alone; (ii) those who were diagnosed with a concomitant aneurysm, arteriovenous malformation, moyamoya disease or haematological system diseases. The Ethics Committee of Nanjing First Hospital and Jinling Hospital approved the study. All patients gave their informed consent before their inclusion in the study.

### Sample size calculation and data collection

The sample size was calculated using the area under the receiver operator characteristic curve. An expected area under the curve value was 0.70. The *α*‐level was set as 0.05. The power was set to 80%. According to our previous study, the sICH rate was 15.0% [5]. Assuming the follow‐up imaging data for haemorrhage identification were absent in 5% of the patients, the total sample size was estimated to be approximately 320.

After admission, data concerning patient characteristics, vascular risk factors, radiological, surgery features and laboratory findings were collected. Baseline neurological deficit was measured by the National Institutes of Health Stroke Scale (NIHSS) score [17]. Infarct volume was assessed using the Alberta Stroke Program Early CT Score (ASPECTS). ASPECTS is a 10‐point quantitative scoring system based on the extent of ischaemic damage within defined regions of the middle cerebral artery territory [18]. Stroke aetiology was categorized according to the criteria of Trial of Org 10172 in Acute Stroke Treatment (TOAST) [19]. Collateral status was evaluated by the American Society of Interventional and Therapeutic Neuroradiology/Society of Interventional Radiology grading system, with grades 0–1 indicating poor collateral circulation [20]. Successful reperfusion was defined as modified Thrombolysis in Cerebral Infarction grade 2b−3 [6]. Onset‐to‐recanalization time was defined as the duration from stroke onset to the first digital subtracted angiography series, indicating recanalization of the occluded artery.

### Determination of sICH and non‐sICH

Computed tomography scans were typically performed 24–72 h post‐EVT or upon the manifestation of clinical symptoms suggestive of intracranial haemorrhage (ICH) to confirm its presence. According to the Heidelberg Bleeding Classification [21, 22], ICH after treatment of ischaemic stroke can manifest as subdural, subarachnoid, intraventricular and intracerebral haemorrhage, either within or outside of brain ischaemic lesions. The sICH was diagnosed if a new ICH was associated with any of the following conditions: (i) increase of ≥4 points in total NIHSS score at the time of diagnosis compared with immediately before worsening or ≥2 points in one NIHSS category; (ii) ICH led to intubation, hemicraniectomy, ventricular drain placement or other major medical/surgical intervention. All radiological data underwent consensus readings by two trained neurologists in a double‐blind manner. The non‐sICH group consisted of individuals with asymptomatic ICH and those without any evidence of ICH.

### Measurement of Cav‐1 level

Blood samples were collected post‐EVT from each subject, typically within 24 h of stroke onset. Serum Cav‐1 concentration was measured by enzyme‐linked immunosorbent assay (ELISA) kit (Human Cav‐1 ELISA Kit, Cat# EK1494, Sabbiotech, USA) according to the manufacturer's instructions.

### Statistical analysis

A two‐sided *p* value <0.05 was considered statistically significant in all analyses. The data were analysed using R software (version 4.3.1; Vienna, Austria). Continuous variables were demonstrated as mean ± standard deviation or median with the interquartile range (IQR). The Shapiro–Wilk test was employed to analyse the normal distribution of the data. Student's *t* test was utilized if continuous variables were normally distributed or a nonparametric Mann–Whitney *U* test was performed if not. Categorical variables were expressed as percentages and compared using chi‐squared tests or Fisher's exact tests.

Multivariable regression logistic analysis was used to evaluate the independent effect of Cav‐1 level on sICH. Model 1 was adjusted for demographic characteristics. Model 2 was adjusted for demographic characteristics, hypertension, baseline NIHSS score, pre‐treatment ASPECTS, poor collateral status, successful reperfusion and baseline glucose levels. The pattern and magnitude of the association of Cav‐1 level with sICH was also evaluated using a logistic regression model with restricted cubic splines with three knots (at 5th, 50th and 95th percentiles) adjusted for covariates included in model 2 [23]. Furthermore, the net reclassification index and integrated discrimination improvement were utilized to explore the predictive value of adding the Cav‐1 level to the conventional risk factors model.

## RESULTS

### Baseline characteristics

Throughout the study, 354 anterior circulation large artery occlusive stroke patients treated with EVT were screened. A total of 29 patients were excluded from this study, including 18 patients treated without a stent‐like retriever or aspiration system, seven patients diagnosed with a concomitant aneurysm, arteriovenous malformation, moyamoya disease or haematological system diseases, and four patients without follow‐up imaging data for haemorrhage identification. Finally, a cohort of 325 patients (63.7% male, mean age 68.6 ± 12.3 years) were recruited for the final analysis. The median level of serum Cav‐1 was 0.186 ng/mL (IQR 0.097–0.282). The main patient characteristics are shown in Table 1. The median NIHSS score was 13.0 (IQR 10.0–17.0), and the median ASPECTS was 9.0 (IQR 8.0–9.0). The median time from onset to recanalization was 362.0 min (IQR 254.5–555.0). Before the EVT, 139 (42.8%) patients received intravenous thrombolysis. A total of 159 patients (48.9%) showed poor collateral status at baseline, whilst 277 patients (85.2%) obtained successful reperfusion.

**TABLE 1:** Baseline characteristics of ischaemic stroke patients with sICH and without sICH.

Variables	All patients (*n* = 325)	With sICH (*n* = 47)	Without sICH (*n* = 278)	*p* value
Demographic characteristics				
Age, years	68.6 ± 12.3	67.3 ± 13.6	68.8 ± 12.1	0.434
Male, *n* (%)	207 (63.7)	30 (63.8)	177 (63.7)	0.983
Medical history, *n* (%)				
Hypertension	228 (70.2)	33 (70.2)	195 (70.1)	0.992
Diabetes mellitus	79 (24.3)	7 (14.9)	72 (25.2)	0.104
Hyperlipidaemia	32 (9.8)	4 (8.5)	28 (10.1)	0.740
Current smoker	126 (38.8)	16 (34.0)	110 (39.6)	0.472
Coronary heart disease	49 (15.1)	7 (14.9)	42 (15.1)	0.970
Systolic blood pressure, mmHg	137.0 ± 23.4	139.3 ± 22.1	136.6 ± 23.7	0.472
Diastolic blood pressure, mmHg	81.9 ± 14.2	83.7 ± 14.7	81.6 ± 14.1	0.361
Time from onset to blood draw, h	19.0 (13.0, 24.0)	18.5 (13.0, 24.0)	19.0 (13.0, 23.0)	0.881
Time from onset to recanalization, min	362.0 (254.5, 555.0)	381.0 (302.0, 472.5)	360.0 (250.0, 576.0)	0.749
Baseline NIHSS, score	13.0 (10.0, 17.0)	16.0 (12.0, 20.0)	13.0 (1.0, 16.0)	0.003
Baseline ASPECTS, score	9.0 (8.0, 9.0)	8.0 (7.0, 9.0)	9.0 (5.0, 9.0)	0.001
Stroke subtypes, *n* (%)				0.499
Large arterial atherosclerosis	158 (48.6)	20 (42.6)	138 (49.6)	
Cardioembolism	138 (42.5)	21 (44.7)	117 (42.1)	
Others/unknown	29 (8.9)	6 (12.8)	33 (8.3)	
Administering IVT before EVT, *n* (%)	139 (42.8)	23 (48.9)	116 (41.7)	0.356
Poor collateral status, *n* (%)	159 (48.9)	29 (61.7)	130 (46.8)	0.048
Successful recanalization, *n* (%)	277 (85.2)	36 (76.6)	241 (86.7)	0.071
The occluded vessel, *n* (%)				0.827
Internal carotid artery	113 (34.8)	17 (36.2)	96 (34.5)	
Middle cerebral artery	212 (65.2)	30 (63.8)	182 (65.5)	
Laboratory data				
Baseline blood glucose, mmol/L	7.5 ± 3.1	8.6 ± 3.3	7.3 ± 3.0	0.009
Hs‐CRP, mg/L	10.6 (3.6, 23.8)	8.7 (3.9, 30.5)	10.6 (3.5, 23.3)	0.998
Caveolin‐1, ng/mL	0.186 (0.097, 0.282)	0.130 (0.053, 0.217)	0.191 (0.108, 0.298)	0.011

Abbreviations: ASPECTS, Alberta Stroke Program Early Computed Tomography Score; EVT, endovascular thrombectomy; Hs‐CRP, hyper‐sensitive C‐reactive protein; IVT, intravenous thrombolysis; NIHSS, National Institutes of Health Stroke Scale; sICH, symptomatic intracranial haemorrhage.

### Factors associated with sICH after EVT

According to the Heidelberg Bleeding Classification, 47 patients (14.5%) were classified with sICH within 72 h after mechanical thrombectomy treatment. Compared to the non‐sICH group, the sICH group had a higher poor collateral status rate (61.7% vs. 46.8%; *p* = 0.048) and baseline blood glucose level (mean 8.6 vs. 7.3 mmol/L; *p* = 0.009). Patients with sICH had lower baseline ASPECT scores (median 8.0 vs. 9.0; *p* = 0.001) and higher baseline NIHSS scores (median 16.0 vs. 13.0; *p* = 0.001). Moreover, the sICH group exhibited a decreased level of Cav‐1 compared to the non‐sICH group (median 0.130 vs. 0.191 ng/mL; *p* = 0.011).

### Association of Cav‐1 level with sICH

**FIGURE 1:**
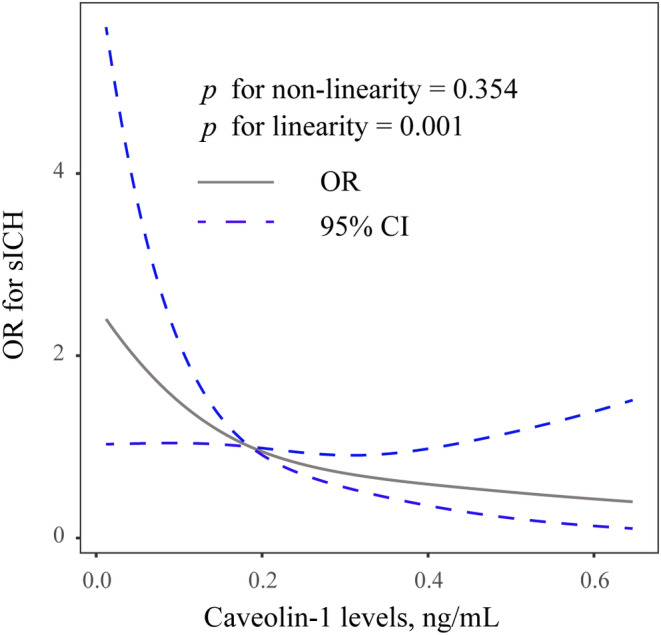
Correlation between Cav‐1 levels and risk of sICH. Odds ratio (OR) and 95% confidence interval (CI) derived from restricted cubic spline regression with three knots (at 5th, 50th and 95th percentiles). The odds ratio was controlled for the same variables as model 2 in Table 2. sICH, symptomatic intracranial haemorrhage.

After multivariable adjustment for variables with a *p* value <0.1 in univariate analysis, decreased serum Cav‐1 level was significantly associated with a higher risk of sICH (first quartile vs. fourth quartile, odds ratio 3.077; 95% confidence interval 1.128–8.393; *p* = 0.028) (Table 2, model 2). The observed association remained significant when Cav‐1 concentration was analysed as a continuous variable. Also, multivariable‐adjusted spline regression models showed a linear dose–response association between serum Cav‐1 and sICH (*p* for nonlinearity 0.354; *p* for linearity 0.001; Figure 1). Furthermore, the inclusion of Cav‐1 into models 1 and 2 modestly improved the net reclassification index and integrated discrimination improvement for sICH prediction (Table 3).

**TABLE 2:** Multivariate regression analysis for the association between circulating caveolin‐1 levels and sICH.

Variables	Unadjusted model		Model 1		Model 2	
	OR (95% CI)	*p* value	OR (95% CI)	*p* value	OR (95% CI)	*p* value
Caveolin‐1, per 1‐unit increase	0.065 (0.006–0.720)	0.026	0.057 (0.005–0.642)	0.029	0.055 (0.005–0.669)	0.038
Caveolin‐1, quartile						
1st	3.356 (1.332–8.455)	0.011	3.624 (1.424–9.224)	0.011	3.077 (1.128–8.393)	0.028
2nd	1.303 (0.461–3.685)	0.617	1.297 (0.456–3.687)	0.626	1.334 (0.433–4.115)	0.661
3rd	1.710 (0.627–4.633)	0.295	1.744 (0.638–4.768)	0.278	1.364 (0.461–4.040)	0.574
4th	Reference		Reference		Reference	

*Note*: Model 1, adjusted for demographic characteristics; model 2, adjusted for demographic characteristics, hypertension, baseline NIHSS score, pre‐treatment ASPECTS, poor collateral status, successful reperfusion and baseline glucose levels.

Abbreviations: ASPECTS, Alberta Stroke Program Early Computed Tomography Score; CI, confidence interval; NIHSS, National Institutes of Health Stroke Scale; OR, odds ratio; sICH, symptomatic intracranial haemorrhage.

**TABLE 3:** Reclassification statistics (95% CI) for sICH after adding caveolin‐1 levels.

Models	NRI (continuous)		IDI	
	Estimate (95% CI)	*p* value	Estimate (95% CI)	*p* value
Model 1				
+Caveolin‐1 levels (continuous)	0.241 (−0.058–0.539)	0.114	0.022 (0.007–0.037)	0.004
+Caveolin‐1 levels (quartile)	0.254 (−0.046–0.577)	0.097	0.024 (0.007–0.041)	0.007
Model 2				
+Caveolin‐1 levels (continuous)	0.397 (0.106–0.689)	0.007	0.027 (0.020–0.045)	0.002
+Caveolin‐1 levels (quartile)	0.320 (0.109–0.622)	0.037	0.026 (0.001–0.040)	0.041

*Note*: Model 1, adjusted for demographic characteristics; model 2, adjusted for demographic characteristics, hypertension, baseline NIHSS score, pre‐treatment ASPECTS, poor collateral status successful reperfusion and baseline glucose levels.

Abbreviations: ASPECTS, Alberta Stroke Program Early Computed Tomography Score; CI, confidence interval; IDI, integrated discrimination improvement; NIHSS, National Institutes of Health Stroke Scale; NRI, net reclassification improvement; sICH, symptomatic intracranial haemorrhage.

## DISCUSSION

The main finding of this study is that serum Cav‐1 was an independent predictor of sICH in acute ischaemic stroke treated with EVT. However, it is unknown at present whether additional evaluation and treatment of patients with decreased Cav‐1 could result in a decrease in sICH incidence with improved prognosis, which suggests future research directions.

It was found that 14.5% of patients experienced sICH in our stroke cohort, which was slightly higher than the data obtained from populations of white dominance [24, 25]. Prior available studies on sICH used inconsistent definitions and ethnic groups, leading to discrepancies in incidence rates and associated factors. No difference was found between patients with and without sICH regarding age, sex, risk factors, baseline blood pressure, aetiology, prior thrombolytic therapy, time from onset to recanalization and site of the occluded vessel. It was observed that the associated factors for sICH were baseline NIHSS score, ASPECTS, collateral status and baseline blood glucose, which were also substantiated by previous reports [5, 7, 22]. These findings can provide information for clinicians treating patients in an acute vascular setting.

Caveolin‐1 is the principal structural protein forming the caveolar domains. It has been implicated in maintaining blood–brain barrier permeability, inhibiting oxidative stress and counteracting neuroinflammatory processes [8, 26]. Some population‐based studies have established the association between Cav‐1 and cardiovascular disease [15, 16, 27, 28]. Our previous study confirmed that reduced Cav‐1 was associated with poor functional outcomes at 3 months in ischaemic stroke patients after EVT, amongst which existed a negative linear dose–response association [27]. Circulating Cav‐1 concentration was positively correlated to cerebral microbleeds in acute ischaemic stroke patients [15], suggesting the role of Cav‐1 in vascular endothelial dysfunction. In further support of this, investigators found that patients with sICH have lower Cav‐1 levels after tissue plasminogen activator treatment [16]. Based on our cohort of patients, preliminary evidence was provided that reduced serum Cav‐1 was remarkably associated with an increased risk of sICH after EVT. The association was independent of established cardiovascular risk factors and stroke severity, pre‐treatment infarct volume, collateral status, successful reperfusion and baseline glucose levels.

Several potential biological mechanisms could explain the observed effect of circulating Cav‐1 on the presence of sICH after reperfusion treatment. A first rational approach is that lower circulating Cav‐1 concentration may be associated with decreased Cav‐1 expression on endothelial cells in the ischaemic brain tissue. Reduced Cav‐1 would cause increased permeability of the blood–brain barrier [12, 29, 30], which serves as a key pathological factor contributing to sICH. Another potential explanation is that Cav‐1 is involved in the regulation of vascular thrombo‐inflammation [12] and mitochondrial redox homeostasis [32]. Downregulation of Cav‐1 was found to amplify proinflammatory cytokine production, including interleukin‐1β, monocyte chemoattractant protein 1 and vascular cell adhesion molecule 1 [31]. Our previous study confirmed that specific up‐expression of Cav‐1 in the endothelium using the adeno‐associated virus markedly suppressed endothelial inflammatory activation, immune cell infiltration and microthrombosis formation in the ischaemic penumbra area [12]. Importantly, Cav‐1 has been reported to play a vital role in collateral vessel remodelling [33], a compensatory process with potential prognostic benefits. Further studies are needed to explore the precise mechanism of Cav‐1 underlying sICH after reperfusion therapy.

The strengths of our study lie in the prospective design and the recruitment of a relatively large population of ischaemic stroke patients with large‐vessel occlusion in the anterior circulation. However, the findings of the present study should be interpreted with caution due to the following limitations. First, only a single blood sample was collected within 24 h of admission, preventing more information being collected on potential fluctuations in Cav‐1 level over time. Secondly, due to the time‐consuming nature and the potential for cross‐reactivity of ELISA, serum Cav‐1 quantification may not be suitable for urgent decision‐making. Thirdly, a survival bias cannot be excluded: the patients who died before the enrolment may have a higher probability of sICH. This bias would induce an underestimation of the reported association. Finally, the observational nature of our study could hinder the generalization of our results. Therefore, it remains to be established whether these results can be generalized to other ethnic groups.

In conclusion, our study demonstrated that lower levels of Cav‐1 were correlated with an aggravated risk of sICH in large‐vessel occlusive stroke patients who undergo EVT. Further studies from various populations are needed to replicate our findings.

## AUTHOR CONTRIBUTIONS

**Yi Xie:** Conceptualization; methodology; investigation; validation; software; data curation; writing – original draft; formal analysis; funding acquisition. **Min Wu:** Conceptualization; methodology; formal analysis; writing – original draft; investigation. **Yun Li:** Data curation; validation; investigation. **Ying Zhao:** Investigation; validation; data curation; supervision. **Shuaiyu Chen:** Methodology; validation; investigation; data curation. **E. Yan:** Data curation; supervision. **Zhihang Huang:** Validation; data curation; resources. **Mengdi Xie:** Investigation; visualization; validation. **Kang Yuan:** Investigation; validation. **Chunhua Qin:** Conceptualization; methodology; writing – review and editing. **Xiaohao Zhang:** Conceptualization; methodology; funding acquisition; writing – review and editing; supervision; project administration.

## FUNDING INFORMATION

This study was partly supported by the National Natural Science Foundation of China (No. 82371311; No. 82171331), China Postdoctoral Science Foundation (No. 2023M731746), National Science Foundation of Jiangsu Province (No. BK20221553), Nanjing Medical Science and Technology Development Foundation for Distinguished Young Scholars (JQX23006) and the Future Science and Technology Talent Program of Nanjing First Hospital.

## CONFLICT OF INTEREST STATEMENT

All the authors declare that there is no conflict of interest.

## Data Availability

The data that support the findings of this study are available on request from the corresponding author.
